# How to Protect Medical Staff in the COVID-19 Battlefield After Work

**DOI:** 10.3389/fpubh.2020.00421

**Published:** 2020-08-06

**Authors:** Xiaojie Huang, Jina Li, Hengxing Liang, Chen Chen

**Affiliations:** ^1^Department of Cardiovascular Surgery, The Second Xiangya Hospital, Central South University, Changsha, China; ^2^Department of Thoracic Surgery, The Second Xiangya Hospital, Central South University, Changsha, China

**Keywords:** coronavirus disease 2019, self-quarantine, facial masks, global pandemic, medical staffs

Protection of health care professionals is of great importance and concern during the coronavirus disease 2019 (COVID-19) pandemic. Previous papers have given recommendations of protection of medical staff while working in hospitals and clinics facing the new disease (1–4), but what was recommended after they left work and returned to their residences was barely mentioned. Actually, it is crucial for the protection for medical staff themselves, their families, and also public health. We hereby share our experience about the designed after-work protocols that protected 482 medical staff that were sent from Changsha to Wuhan and to a locally designated hospital to support the medical system from the highly contagious COVID-19.

In China, as close contacts of the suspicious or confirmed COVID-19 patients, most of the medical staff that worked in the COVID-19 clinics/wards lived in designated hotels or temporary residences so that necessary self-quarantine was guaranteed and the superfluous risk of transmission of infection was minimized as much as possible. We believe this article about the protocols we designed and strictly followed based on aseptic principles and quarantine regulations would also be of some help for those medical professionals who return home after work.

## Area Setting

**Figure 1:**
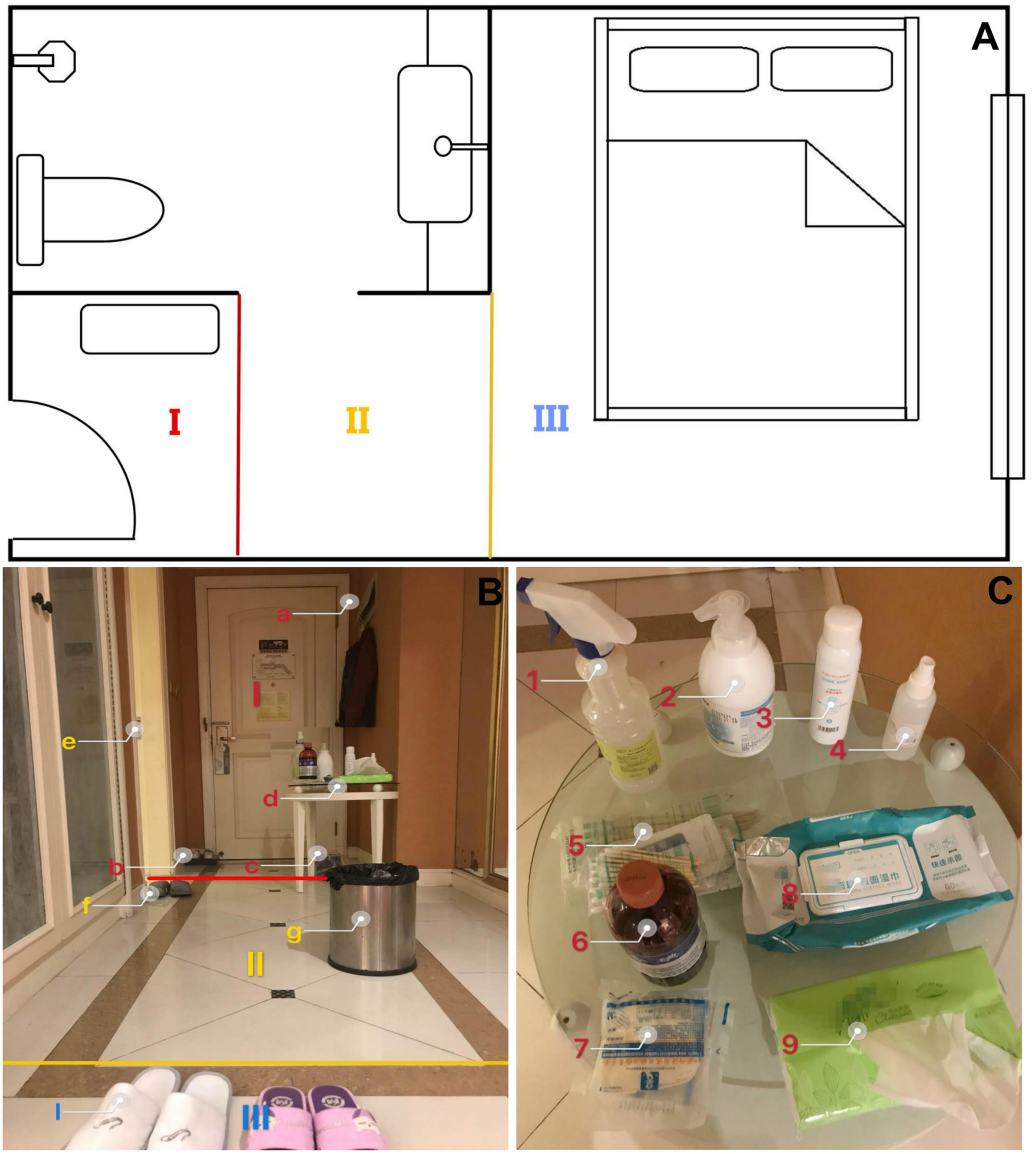
**(A)** 2-D floor plan and the area setting. (I) Contaminated zone; (II) Buffer zone; (III) Clean zone. **(B)** 3-D floor plan and the area setting. I. Contaminated zone: (a) Coat hangers, (b) shoe box with a disposable plastic bag with chlorine-containing disinfectant, (c) garbage bags and the garbage can, (d) a small shelf; II. Buffer zone: (e) bathroom, (f) shoes for buffer zone, (g) garbage can; III. Clean zone: (i) Indoor shoes; **(C)** In the contaminated zone a shelf was set for: (1, 2) hand disinfection solution, (3) chlorine-containing disinfectant, (4) alcoholic sprinkling can, (5) cotton swabs and gauze, (6) iodine, (7) gloves, (8) alcohol pads, and (9) tissue.

An infection control area was set immediately after the entrance of the residences, divided into the contaminated area, buffer area, and clean area (Figures 1A,B).

### Contaminated Zone

First area after the entry. A shoe box with a disposable plastic bag with chlorine-containing disinfectant was set there to get the shoe soles sterilized. The plastic bag was replaced daily by the resident. It is extremely recommended considering that our colleagues in Wuhan found that that about half of healthcare professionals working in intensive care units carried coronavirus on the soles of their shoes. (5) Coat hangers were set behind the door for outside clothes. A shelf was set for a hand disinfection solution, alcoholic sprinkling can, alcohol pads, tissues, cotton swabs, gauze, gloves, garbage bags, and a garbage can (Figure 1C).

### Buffer Zone

The showers and bathrooms; for shower slippers, toiletries, alcoholic pads, Q-tips, and one trash can.

### Clean Zone

The rest of the indoor living area. Indoor shoes and clothes were required.

## Protocols

Step 1: Enter the residence (hotel rooms or home), take off and put the shoes in the shoe box for sterilization; hand hygiene; use alcoholic pads to disinfect the smart phone and spectacle frames; take off and disinfect the outside clothes with the sprinkling can and place them on the hanger.
Step 2: Enter the bathroom, wash hands again; take a shower; use alcoholic pads for auricles and ala nasi.
Step 3: Put on indoor clothes and shoes and get into the clean area.

## Tips

### Facial Masks

We strongly recommend the daily wear of masks for medical staff. We do believe it is of great importance for public health. DO NOT try to sterilize the facial masks with alcohol even if replacement is not optional. Alcohol would damage the waterproof layer of facial masks and impair the protection efficacy. When repeated use of facial masks is inevitable, we recommend processing the facial mask with the heat of a hairdryer for 30 min to hopefully deactivate the viruses (4, 6).

### Garbage Disposal

Indoor garbage was put in sealed garbage bags by the residents themselves. Bags were placed outside the door or designated sites and specially labeled as a reminder for special handling by the staff. Professionally trained workers of the hotels would collect the labeled bags daily.

### Re-entry to the Room

Going out of the rooms to get food and living supplies and re-entry are somewhat inevitable. Follow the protocols and skip Step 2 if clinics/wards are not where you come back from.

As of this article's writing, no confirmed case of COVID-19 has been reported among our medical staff; 482 medical staff were tested negative with CT scan, swab-gold pharyngeal, and serology testing. Considering the difficulty in identifying asymptomatic carriers, we believe it is crucial to pay attention to all the details in self-protection. After all, health care professionals are the most valuable resources in the global pandemic. Sincerely, we hope our experience will be of help to medical staff worldwide.

## Author Contributions

CC provide the concepts and ideas of the article. XH and JL drafted the paper. HL and CC performed a critical revision of the first draft and the final editing of the manuscript. All authors have critically revised the manuscript for important intellectual content and gave final approval for the version to be published.

## Conflict of Interest

The authors declare that the research was conducted in the absence of any commercial or financial relationships that could be construed as a potential conflict of interest.
